# Molecular mechanisms underlying the impact of mutations in SOD1 on its conformational properties associated with amyotrophic lateral sclerosis as revealed with molecular modelling

**DOI:** 10.1186/s12900-018-0080-9

**Published:** 2018-02-05

**Authors:** Nikolay A. Alemasov, Nikita V. Ivanisenko, Srinivasan Ramachandran, Vladimir A. Ivanisenko

**Affiliations:** 1 The Federal Research Center Institute of Cytology and Genetics, The Siberian Branch of the Russian Academy of Sciences, 630090 Novosibirsk, Russia; 2 Novosibirsk State University, 630090 Novosibirsk, Russia; 3 Functional Genomics Unit, Council of Scientific and Industrial Research-Institute of Genomics and Integrative Biology (CSIR-IGIB), South Campus, New Delhi, 110025 India; 4 Academy of Scientific and Innovative Research, CSIR-IGIB, South Campus, New Delhi, 110025 India

**Keywords:** ALS, SOD1, Misfolding, Aggregates, Elastic networks, Hydrogen bonds, Copper

## Abstract

**Background:**

So far, little is known about the molecular mechanisms of amyotrophic lateral sclerosis onset and progression caused by *SOD1* mutations. One of the hypotheses is based on SOD1 misfolding resulting from mutations and subsequent deposition of its cytotoxic aggregates. This hypothesis is complicated by the fact that known SOD1 mutations of similar clinical effect could be distributed over the whole protein structure.

**Results:**

In this work, a measure of hydrogen bond stability in conformational states was studied with elastic network analysis of 35 SOD1 mutants. Twenty-eight hydrogen bonds were detected in nine of 35 mutants with their stability being significantly different from that with the wild-type. These hydrogen bonds were formed by the amino acid residues known from the literature to be located in contact between SOD1 aggregates. Additionally, residues disposed between copper binding sites of both protein subunits were found from the models to form a stiff core, which can be involved in mechanical impulse transduction between these active centres.

**Conclusions:**

The modelling highlights that both stability of the copper binding site and stability of the dimer can play an important role in ALS progression.

**Electronic supplementary material:**

The online version of this article (10.1186/s12900-018-0080-9) contains supplementary material, which is available to authorized users.

## Background

Amyotrophic lateral sclerosis (ALS) is a fatal neurodegenerative disease which manifests in two forms: familial and sporadic [1]. The second most prevalent and studied cause of familial ALS is mutations in the *SOD1* gene, which codes for the superoxide dismutase-1 enzyme [2]. Currently, a number of hypotheses are suggested as to the mechanism for disease progression at the molecular level [1, 2]. One posits an aggregation of misfolded SOD1 proteins caused by mutations [2–4].

SOD1 consists of two of the same monomers of 153 amino acid residues, each of which is a beta-barrel composed of eight anti-parallel beta-sheets [5, 6]. All known SOD1 mutations are divided into two groups: wild-type (WT)-like and mutations in metal binding site regions [7, 8]. Zinc and copper ions are known to stabilize SOD1’s structure [9–11]. Inter-subunit interface destabilization makes SOD1 monomerization more probable and, thus, can lead to protein aggregation [12, 13]. Experimental studies of SOD1 aggregation are typically performed on animal model lines under conditions of overexpression of mutant proteins. This complicates defining the actual mechanisms of protein aggregation [14]. Another issue is that the positions with known SOD1 mutations of similar clinical effect are distributed over the whole protein’s structure [7, 15]. Therefore, it remains unclear how mutations in different SOD1 regions can lead to similar outer effects, i.e., aggregation. A range of investigations have been conducted both in vitro [10, 16, 17] and in silico [9, 13, 15, 18–22] aimed at uncovering the mechanisms of SOD1 aggregation and ALS progression. These studies have analysed the impact of a particular mutation in the protein on disease progression. However, there is a definite lack of studies with the objective of combining structural and dynamic consequences resulting from each of the SOD1 mutations known to result in ALS. What changes take place in the mutant structures that bring about the same effect, i.e., disease, though being manifested to various extents?

Hydrogen bonds are known to play an important role, in particular, in stabilizing protein structure [23–26]. One of in silico investigations of SOD1 structure and dynamics already carried out an analysis of the hydrogen bonds formed inside the protein [13]. Recently, the influence of 39 mutations of SOD1 on its structure was studied using molecular dynamics (MD) simulations [27]. Hydrogen bonds were identified with the percentage of time in the MD trajectory being formed (or their stability) found to be significantly correlated with ALS patients survival time. In the current work, SOD1 dynamics modelling was performed with elastic network (EN) models [28]. The modelling with EN has also been conducted by other authors, especially for docking [29], protein intermediate-state detection [30], and investigating evolutionary aspects of protein functioning [31]. This approach has also been applied to study large-scale protein dynamics that are challenging for MD [32–36].

In the present study, two similar measures of hydrogen bond stability were applied to analyse the differences between conformational properties of SOD1 mutants and the WT. The first was calculated from MD [27] and represents time average stability of hydrogen bonds (STA), while the second was obtained using EN modelling and reflects the ensemble average stability (SEA). While STA describes a percentage of MD simulation time when the bond exists, SEA represents a ratio of the count of conformations with the bond formed to the total count of conformations obtained in an EN simulation. The goal here was to unravel the molecular mechanisms linking SOD1 mutations with its conformational features that might be significant for its aggregation and consequently ALS progression. For this to be carried out, EN modelling of mutant SOD1s was performed followed by detection of hydrogen bonds with their SEA being different across mutant structures versus the WT. Mechanical stiffness of SOD1 structure was evaluated, and the protein’s regions with increased stiffness were established. These regions were found to effectively transmit a mechanical perturbation from the surface of the protein to crucial for its stability sites. As a result of the analysis, the structure and dynamics of SOD1 mutants were observed to feature a common mechanism of disruption of the protein’s conformational properties associated with ALS.

## Methods

### Simulation

The study was performed on the wild-type SOD1 and the following 35 mutants taken from the ALSOD database (http://alsod.iop.kcl.ac.uk/): A4V, C6G, V7E, L8Q, G10 V, G12R, F20C, G37R, L38 V, G41D, G41S, H43R, H46R, H48Q, D76V, L84F, L84 V, G85R, N86 K, A89V, D90A, G93R, E100G, D101N, S105 L, L106 V, I112M, I112T, G114A, D124V, D125H, G127R, N139H, L144S, and V148I.

Spatial structures of the 35 mutant SOD1s were obtained by introduction of mutations into the human WT SOD1 protein structure (PDBID: 2V0A) using FoldX software [37]. As a reference structure, PDBID 2V0A of the WT SOD1 was employed. Mutant structure refinement, including removal of clashes and optimization of amino acid side-chain orientations, was completed with FoldX, as well. The structure corresponding to each of these conformations was subsequently minimized using the AMBER 12 software suite [38] in order to avoid physically inaccessible torsion angles, bond lengths, and other structural properties.

The WT and 35 mutant structures were modelled with EN modelling using ElNemo with the default parameters set [35]. The number of lowest frequency normal modes to be computed (NMODES) was set to 5. Minimum, maximum perturbation parameters (DQMIN/DQMAX) and step size between DQMIN and DQMAX (DQSTEP) were set to − 100, 100 and 20, respectively. The number of residues to be grouped together by diagrtb (NRBL) was set to “auto”, meaning that this number would be determined automatically as a function of protein size to optimize computation speed. The cut-off employed to identify elastic interactions (CUTOFF) was set to 8. Each elastic model was provided with an ensemble of 11 conformations for each of five non-trivial modes with the highest collectivity measure. Thus, in total, 55 conformations were obtained for the WT and every SOD1 mutant, representing large-scale fluctuations of these molecules’ structure.

As a measure of the EN simulation validity, a correlation among eigenvectors for the same vibration modes was calculated for the WT and SOD1 mutants studied. All these correlation coefficients appeared to be within a 0.97 to 0.99 interval, indicating they were structural changes induced by introduction of amino acid substitutions that resulted in the difference in the eigenvectors’ components, and not an alternative order of the corresponding eigenvalues.

### Analysis

A table of hydrogen bonds was constructed for the WT SOD1 and its mutants [see Additional file 1]. To this end, hydrogen bonds were detected by the cpptraj utility for each of the 55 conformations analysed for the WT and mutants using AmberTools 13 [39]. Then, the SEA of every single hydrogen bond found was calculated within the conformation ensemble given as ${SEA}_i^m=\frac{N_i^m}{55}$, where ${N}_i^m$ is a number of conformations with the *i*^th^ hydrogen bond being formed in mutant *m*, and 55 stands for a total number of conformations for the mutant. Columns of this table corresponded to the SOD1 structures, including the WT and mutants. Its rows feature the hydrogen bonds detected. Each cell of the table contains the SEA averaged over 55 conformations.

Mutant SOD1s were clustered by the SEA of their hydrogen bonds through the mean-shift method [40] via the sklearn package for Python [41]. The *bandwidth* parameter for this clustering, which influenced the number of clusters found, was estimated using the internal procedure of the sklearn implementation of the method.

The mechanical stiffness between the amino acid positions in the protein structure was calculated via EN modelling by the anisotropic network model (ANM) approach [42, 43]. Mechanical stiffness between a pair of amino acid residues is expressed as an equivalent spring stiffness constant, defined by the cosine between the direction of external force applied and the off-set direction of the residues corresponding to the vibration mode within an elastic model [42]. In other words, the higher the constant, the stiffer the supposed spring and the greater the mechanical stiffness between this pair of residues.

When conducting multiple comparisons, a 5% false discovery rate was utilized [44].

Evolutionary conservation of residues in the protein was obtained via ConSurf with a default setup [45]. As a reference structure, chain A of the X-ray structure PDBID: 2V0A was employed. As well, an automated homolog search with the HMMER algorithm (1 iteration, E-value cut-off = 0.0001) in the UNIREF-90 database was carried out. Up to 150 homolog sequences with 35 to 95% identity were selected for use in ConSurf. Multiple alignment was performed using the MAFFT-L-INS-i method. Evolutionary conservation was calculated by selecting the Bayesian method in ConSurf.

### Important SOD1 residues

The positions for the binding sites for zinc (residues: 63, 71, 80, and 83) and copper (residues: 46, 48, 63, and 120) in SOD1 were taken from PDBID: 2V0A [20]. While the protein consists of seven loops [5, 6, 46], four of these loops are thought to be important for SOD1’s structure (PDBID: 2V0A): disulfide (residues: 49–62), zinc-binding (residues: 63–85), “greek-key” (residues: 102–115), and electrostatic (residues: 121–142). The following residues were considered to be the interface between both monomers as predicted by PrISE software [47] and published in Das (2013) [18]: 5, 7, 17, 50–54, 113–115, 148, and 150–153. Additionally, the following residues were regarded as importantas they had been suggested by many authors to be involved in the contact between SOD1 aggregates: 11–15, 24, 26, 91–92, 97–99, 101–104, 109, and 128–131 [48–52].

### Survival time of ALS patients

SOD1 mutations-caused ALS-patient survival time was obtained from literature [53]. These survival times were expressed in years between the disease’s onset and expiry of the patient.

## Results

### Mutant clustering via their hydrogen bond stability

As a result of EN analysis of 36 SOD1s, including 35 of its mutants and the WT, in total, 1980 3D structures were obtained that corresponded to various conformational states (55 for each of the 36 SOD1s). In total, 934 variants of hydrogen bonds were found inside these 36 structures. The table of hydrogen bonds with their SEA measures for all 36 SOD1s is located in an additional table file [see Additional file 1].

Clustering of the WT and mutants was conducted using the mean-shift method based on the SEA of hydrogen bonds formed by these structures according to EN modelling. The *bandwidth* parameter for this clustering was estimated to be 4.95.

**Fig. 1:**
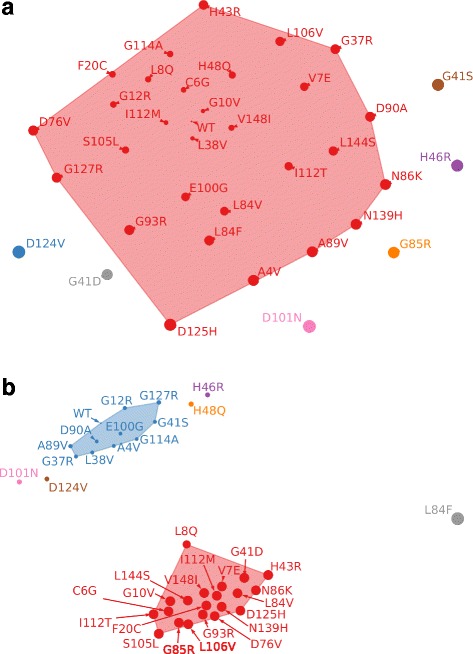
WT SOD1 and its mutants clustered based on (**a**) SEA of their hydrogen bonds; **b** STA of their hydrogen bonds. Radius of circles denotes deviation of hydrogen bonds stability in the corresponding mutant from that in the WT.

All in all, six clusters were established (see Fig. 1a). The same cluster contained the WT and the following mutants: A4V, C6G, V7E, L8Q, G10 V, G12R, F20C, G37R, L38 V, H43R, H48Q, D76V, L84F, L84 V, G85R, N86 K, A89V, D90A, G93R, E100G, S105 L, L106 V, I112T, I112M, G114A, D125H, G127R, N139H, L144S, and V148I. The remaining five mutants (G41D, G41S, H46R, D101N, and D124V) fell into the individual five clusters. Of interest is these five mutants are also among the top ten mutants with the greatest distance to the others in terms of SEA when performing hierarchical clustering [54], which confirmed the mean-shift clustering results [see Additional file 2].

SOD1 mutants clustering using the mean-shift method based on hydrogen bond’s STA was performed in addition to clustering by SEA of their hydrogen bonds formed within EN models. The values of STA were taken from our previous study conducted using MD [27]. As a result of the clustering of SOD1 mutants by STA of their hydrogen bonds using the mean-shift method, seven clusters were identified (see Fig. 1b). The *bandwidth* parameter was estimated to be 1.87. The first two clusters included more than 10 mutants. The remaining five clusters featured five individual mutants (H46R, H48Q, L84F, D101N, and D124V).

### Destructive hydrogen bonds

A further step of the cluster analysis was devoted to searching for hydrogen bonds inside SOD1 mutants with their SEA being most different from inside the WT. Those hydrogen bonds are further referenced as “destructive”. An algorithm to search for these destructive hydrogen bonds includes the following steps:

1. Deviation of SEA of all hydrogen bonds inside mutant *m* from the SEA of the same bonds inside the WT calculated for each of SOD1 mutants studied as ${D}^m=\sqrt{\sum {\left({SEA}_i^m-{SEA}_i^{WT}\right)}^2}$, where ${SEA}_i^m$ is SEA of hydrogen bond *i* in mutant *m*, and ${SEA}_i^{WT}$ is SEA of the same *i*^th^ bond in the WT;
2. Contribution, ${\xi}_j^m$, of the square of the difference between the SEA of hydrogen bond, *j*, inside a mutant *m* and the WT to the *D*^*m*^ of the mutant found as ${\xi}_j^m=\frac{{\left({SEA}_j^m-{SEA}_j^{WT}\right)}^2}{{\left({D}^m\right)}^2}$; and
3. A table H of 35 rows and 9 columns of the ${\xi}_j^m$ value distribution for all hydrogen bonds, *j*, of each mutant *m* was constructed; each cell, *i*, of the H_m_ row featured a count of hydrogen bonds inside the mutant, *m*, with ${\xi}_j^m$ within a range [1/10*(i-1); 1/10*i), where *i* is a cell number ranging from 1 to 9 (see Table 1) [see Additional file 3].

**Table 1:** Fragment of table H

m	H_m_^1^	H_m_^2^	H_m_^3^	H_m_^4^	H_m_^5^–H_m_^9^
A4V	926	0	0	0	0
C6G	920	6	0	0	0
V7E	926	0	0	0	0
L8Q	921	5	0	0	0
G10 V	924	1	0	1	0
L144S	926	0	0	0	0
V148I	924	2	0	0	0

Next, another clustering of SOD1 mutants was performed through the mean-shift by rows, H_m_, of table H. A measure,${\overline{S}}_H=\frac{1}{8}{\sum}_{i=2}^9{H}_m\left[i\right]$, was inspected among the mutants from each cluster detected, where ${\overline{S}}_H$ is a sum of H_m_ cells, *i*, ranging from 2 to 9 and averaged by *i*. This measure, ${\overline{S}}_H$, counts a number of hydrogen bonds {j} with ${\xi}_j^m$ being greater than 10% or, in other words, it tracks the bonds with highest contribution.

Mutants falling inside clusters 2, 3, and 4, in contrast to mutants from cluster 1, were observed to have hydrogen bonds with ${\xi}_j^m$ greater than 10%. Bearing this in mind, mutants F20C and G114A from the third cluster had the highest number of hydrogen bonds with the greatest ${\xi}_j^m$ among other mutants based on the highest S_H_ = 0.94. Hydrogen bonds formed inside mutants of clusters 2, 3, and 4 were investigated in more detail.

**Fig. 2:**
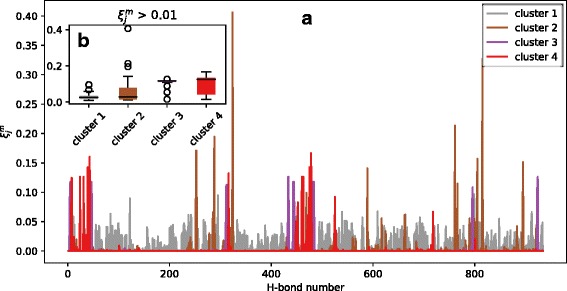
Contribution, ${\xi}_j^m$, of hydrogen bonds inside mutants from each of the clusters to *D*^*m*^. **a** Profile of ${\xi}_j^m$ among all hydrogen bonds for individual clusters; and **b** average ${\xi}_j^m$ over each cluster. Circles correspond to outliers

Mutants from clusters 2, 3, and 4, according to the distribution of SOD1 mutants over clusters (see Table 2), had hydrogen bonds with a significant contribution, ${\xi}_j^m$, to *D*^*m*^ (see Fig. 2). At the same time, mean survival time of patients with these mutations (2.3 years) was below the mean for all mutations. Based on the analysis performed, structures of these nine mutants adopted conformations that were abnormal relative to the WT, and that could be the cause for SOD1 aggregates to be formed, leading to the decreased survival time of patients with these mutations. Mutants with such conformations form hydrogen bonds with their SEA being significantly different from that of the WT.

**Table 2:** SOD1 mutant clustering by H_m_ rows

No	SOD1 mutants	S_H_	ST, years
1	A4V, V7E, G37R, G41S, G41D, H43R, H46R, H48Q, D76V, L84F, L84 V, G85R, N86 K, A89V, D90A, G93R, E100G, D101N, S105 L, L106 V, I112T, D124V, D125H, G127R, N139H, L144S	0.00	5.6 ± 5.4
2	G10 V, L38 V, I112M, V148I	0.31 ± 0.06	2.0 ± 0.7
3	F20C, G114A	0.94 ± 0.06	2.3 ± 0.4
4	C6G, L8Q, G12R	0.67 ± 0.06	2.4 ± 2.2

ST denotes mean survival time of patients with mutations from the corresponding cluster. Standard deviations were given after “±” symbol

**Fig. 3:**
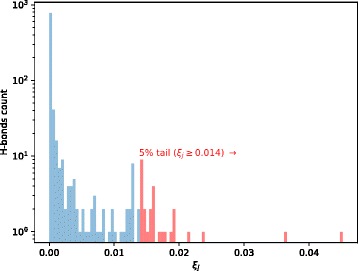
Distribution of mean contributions, ${\overline{\xi}}_j$, of hydrogen bonds inside mutants C6G, L8Q, G10 V, G12R, F20C, L38 V, I112M, G114A, and V148I. Rightmost 5% tail of the distribution is highlighted with red. Note the y-axis has logarithmic scale

Average ${\overline{\xi}}_j=\frac{1}{9}{\sum}_{l=1}^9{\xi}_j^{m_l}$ values were calculated over the hydrogen bonds, *j*, of the nine mutants from clusters 2, 3, and 4 to uncover the most destructive SOD1 hydrogen bonds. Thereafter, the distribution of these ${\overline{\xi}}_j$ values was inspected (see Fig. 3).

**Fig. 4:**
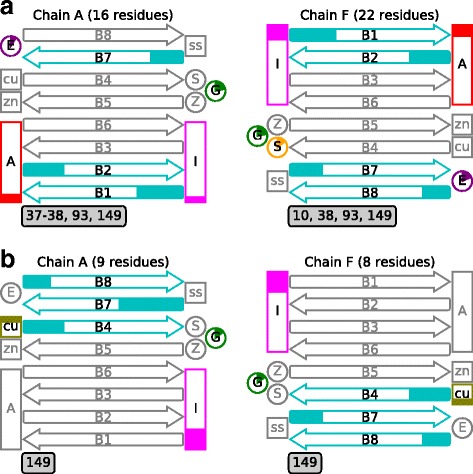
Schematic representation of SOD1 secondary structure with positions of residues (**a**) forming 28 destructive hydrogen bonds that are marked, and (**b**) with most averaged mechanical stiffness, $\overline{K_i}$, highlighted. Chain A is shown on the left and chain F on the right. In parentheses are the number of residues involved in forming hydrogen bonds of a chain. The E, S, Z, and G letters in circles denote electrostatic, disulfide, zinc-binding, and “greek-key” loops, respectively. Letters A and I inside the vertical rectangles indicate those residues involved in the contact of SOD1 aggregates and residues at the interface between subunits, respectively. Labels ss, cu, and zn inside squares stand for residues forming disulfide bond, copper-, and zinc-binding sites, respectively. Horizontal rounded arrows with B1–B8 specify corresponding beta-sheets from 1 to 8. The degree of filling in the circles, squares, and arrows reflects the percentage of residues falling in each element. Numbers below the schemes signify residue positions missed in the markup

As can be seen from Fig. 3, destructive bonds can be defined from rightmost 5% tail of ${\overline{\xi}}_j$ distribution as they are outliers. There were 28 of those bonds with ${\overline{\xi}}_j$ ≥ 0.014, which explain the entire 50% of *D*^*m*^ averaged over mutants of clusters 2, 3, and 4. Amino acid residues 6, 8, 10–12, 14, 16, 18, 37–38, 52–53, 93, 105, 112, 114, 117, 119, 121, 133, 136, 142, 144, and 149 were among those forming those 28 destructive hydrogen bonds. The residues of the 28 destructive hydrogen bonds turned out to be located in beta-sheets 1, 2, 7, and 8, the electrostatic, disulfide, and “greek-key” loops, the dimer interface, and in contact between SOD1 aggregates (see Fig. 4a).

Hydrogen bond STA analysis, as opposed to that with SEA, showed that there were no pronounced differences between the hydrogen bonds’ stability in mutants and the WT. Indeed, STA of hydrogen bonds for all mutants, excepting H48Q, with their contributions, ${\xi}_j^m$, to *D*^*m*^ being less than 10% was observed. There was a hydrogen bond from G10 to V14 (chain A) in H48Q with ${\xi}_j^m$ greater than 10%. Therefore, not a single hydrogen bond could be labelled as destructive based on STA.

### Mechanical stiffness of SOD1 structure

One approach to studying mechanical stiffness between positions of protein amino acid residues is through EN modelling within an ANM [43]. This approach considers a case when a given amino acid residue pair of a protein, represented as an elastic network model, has a force applied towards a line between the centres of the residues [42]. Mechanical stiffness, κ_ij_, between a pair of residues in positions *i* and *j* is expressed in terms of the stiffness constant of an equivalent spring between them. There are a number of studies that have applied mechanical stiffness when investigating flexibility of structurally homologous proteins [55], mechanically characterizing amyloid fibrils [56], modelling entire microtubules [57], exploring the sensitivity of myosin-V to straining forces [58] and determining the mechanical response of guanylate kinase under anisotropic deformations [59], implicating mechanical stiffness as appropriate for probing the mechanical properties of proteins.

Thus, the impact of an external stretching force applied to all pairs of amino acid residues of the protein, and the corresponding mechanical stiffness, was computed from the EN model of WT SOD1. Mean mechanical stiffness, $\overline{K_i}=\frac{1}{153}{\sum}_j^{153}{\kappa}_{ij}$, for given residue in position, *i*, was computed as an average stiffness of all pairs of amino acid residue positions in the protein with this affected position.

Among the positions with the most average stiffness were the following (see Fig. 4b): 46–47, 114–117, and 148–150 in chain A, as well as 46–47, 114–117.F, 148–150 in chain F. These positions were extracted considering their $\overline{K_i}$ from rightmost 0.17%-tail of $\overline{K_i}$ distribution. Fig. 4b portrays how the residues in these positions are part of the copper-binding site, dimer interface, “greek-key” loop, and beta-sheets 4, 7, and 8.

**Fig. 5:**
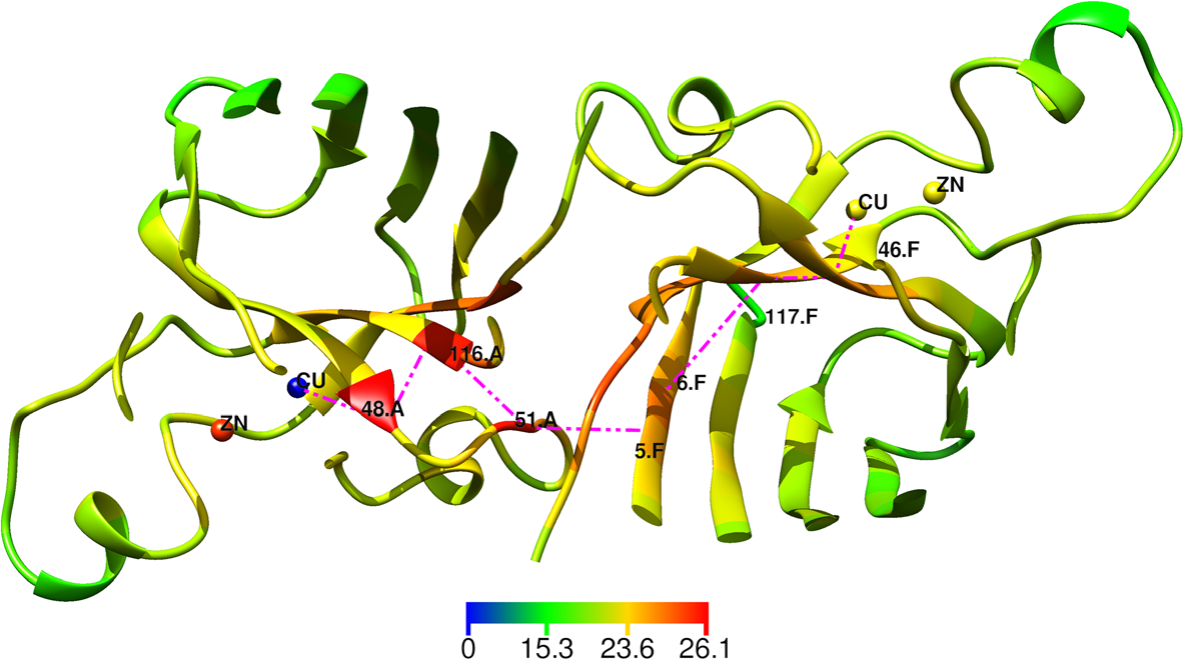
Mechanical stiffness, κ_Cu,i_, between the copper ion of chain A and residues of SOD1. Lines denote a presumable chain of non-covalent interactions between copper-binding sites of both SOD1 monomers. The colour indicates the value of the stiffness

The next step consisted of analysing stiffness κ_Cu,i_, where Cu indicates the copper ion in the structure of SOD1. The most stiff, according to rightmost 0.65%-tail of κ_Cu,i_ distribution, were positions 48, 50–53, 115–116, 118–119, and 146–148 from chain A, and 5–8, 45–47, 117–118, 146–151 from chain F [see Additional file 4]. These positions are located between the copper-binding sites of both SOD1 monomers, comprising a compact fragment of SOD1 spatial structure that can be regarded as an interface of interaction between these active centres (see Fig. 5). The interface consists of the following chain of spatially close amino acid residues which can presumably transmit the mechanical impact between the two copper-binding sites: Cu.A-48.A-116.A-51.A-5.F-6.F-117.F-46.F-Cu.F. The same picture was considering stiffness between the copper ion of chain F and the SOD1 amino acid residues. Indeed, of the highest stiffness were positions: 5–7, 46–47, 117 and 146–151 from chain A, and 48, 51–52, 115–116, 118–119 and 146–148 from chain F [see Additional file 5]. One can see that the presumed path of mechanical impact transmission between these two copper-binding sites was mirrored: Cu.F-48.F-116.F-51.F-5.A-6.A-117.A-46.A-Cu.A.

In order to ascertain what mechanical stiffness the mutations being investigated possess, average stiffness ${\overline{\kappa}}_{m_kc}=\frac{1}{25}{\sum}_{i=1}^{25}{\kappa}_{m_k{c}_i}$ between mutant position, *m*_*k*_, and positions, *c*_*i*_, important for the protein’s structure was calculated for each *m*_*k*_ (k = 1, 32) in chains A and F. Average stiffness characterizes a degree of dependency of movements of a residue from other residues in a protein or, said differently, “the mechanical resistance of individual residues to deformation, in general” [42].

There were 25 residues from metal-binding sites, the dimer interface, and disulfide bonds that are of importance and were considered as such [9–11]. Each set of ${\overline{\kappa}}_{m_kc}$ was compared with a set of κ_ij_ of mechanical stiffness between all pairs of positions in the protein using Kolmogorov-Smirnov non-parametric criterion [60, 61]. The statistical significance of the difference between these two sets obtained for each position, m_k_, of the mutants of SOD1 being studied (k = 1, 32) is shown in an additional table file [see Additional file 6]. Average stiffness, ${\overline{\kappa}}_{m_kc}$, was significantly different (*p* < 0.05) from κ_ij_ for all positions, *m*_*k*_, but 37 and 76, meaning the major mutant positions were remarkable for their stiffness.

The next phase of the analysis of mechanical stiffness was calculating the correlation between survival time of ALS patients with SOD1 mutations and stiffness ${\kappa}_{m_ki}$ where *m*_*k*_ indicated mutant positions (k = 1, 32) and *i* was the position within the protein (*i* = 1, 153). The highest correlation coefficient (*R* = − 0.41, *p* = 0.0217) was for 46th position (see Fig. 5). Noteworthy is this amino acid residue is known to take part in formation of a copper-binding site.

**Fig. 6:**
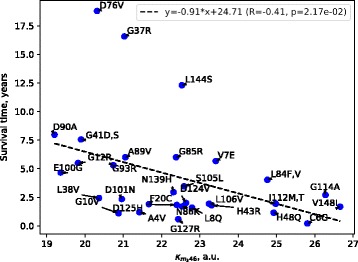
Correlation between survival time of patients with mutations in SOD1 and mechanical stiffness, ${\kappa}_{m_k46}$

Figure 6 depicts mutant positions 37, 76, and 144 dropping out of the relationship. Patients with these mutations have higher survival times than expected according to regression dependence.

### Relationship between mechanical stiffness and evolutionary conservation of amino acid residues

The evolutionary conservation of SOD1 amino acid residues was studied along with mechanical stiffness. Evolutionary conservation was expected to be an additional measure of residues’ importance for the protein’s structure and functioning. The investigation considered a relationship between evolutionary conservation of a given residue in position, *i*, and its mean mechanical stiffness, $\overline{K_i}$.

**Fig. 7:**
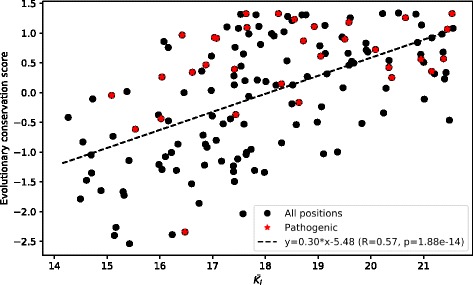
Correlation between mechanical stiffness and evolutionary conservation of SOD1 amino acid residues. Stars denote positions in the protein corresponding to the 35 pathogenic mutations being studied

There was a significant positive correlation between the features mentioned (*R* = 0.57, *p* = 1.88*10^− 14^) (see Fig. 7).

### Identified residue importance for structure and function of the protein

**Fig. 8:**
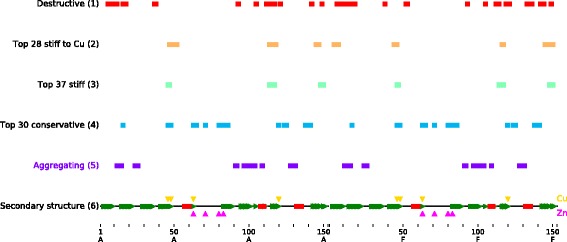
Distribution of the residues identified over SOD1 sequence. (1) Residues forming destructive hydrogen bonds, (2) residues from the interaction interface between copper-binding sites of both SOD1 monomers, (3) residues with top mean mechanical stiffness, (4) top evolutionarily conserved residues, (5) residues from contact between SOD1 aggregates, (6) schematic depiction of SOD1 secondary structure with indications of residues from copper-binding (pink triangles) and zinc-binding (gold triangles) sites

Analysis of SOD1 structural properties, including formation of “destructive” hydrogen bonds and changes in residues’ mechanical stiffness caused by amino acid mutations associated with the pathology was conducted in this investigation through EN. A distribution of the residues identified within the analysis over the SOD1 sequence together with the indication of evolutionarily conserved positions and positions in contact within SOD1 aggregates is depicted in Fig. 8.

Pairwise intersection of lists with positions identified as well as positions from contact between SOD1 aggregates taken from the literature was inspected. The list of residues forming destructive hydrogen bonds turned out to be significantly (*p* = 0.034) intersected with the list of top mean mechanical stiffness residues in the following positions: 114, 117, 149 (chain A); 114, 149 (chain F).

Additionally, pairwise correlation analysis of lists containing residues that were identified was performed. To this end, for each such list, a vector, **v**, of 10 elements was constructed. Each element, v_i_, of the **v** comprised a value equal to a count of residues with positions within range [306/10*(i-1); 306/10*i). As a result, a pair (a, b) of lists of valuable residues was compared using correlations between their corresponding **v**_**a**_ and **v**_**b**_. Thus, the highest correlation was seen between residues forming destructive hydrogen bonds and residues from contact between SOD1 aggregates (*R* = 0.79, *p* = 0.006).

Evolutionary conservation analysis of identified amino acid residues using the Kolmogorov-Smirnov criterion showed that average values of their evolutionary conservation was significantly different from the average evolutionary conservation of all residues in the protein (see Table 3).

**Table 3:** Average evolutionary conservation of amino acid residues important for SOD1 structure and function

Residue list	Mean evolutionary conservations of residues from list	Significance of difference from mean evolutionary conservation over all protein residues
All residues	−1.3*10^−5^ (≈0)	–
Residues forming destructive hydrogen bonds	0.3	0.05
Residues with increased mechanical stiffness to copper	0.57	8.1*10^−4^
Residues with top mean stiffness to all residues	0.64	0.013

## Discussion

When comparing mutant clusters by their hydrogen bond SEA based on the relationship between the thermostability of SOD1 mutants and their clinical effects taken from the literature [16], it was found that mutants from the individual clusters were located in corners on the plane defined by “ΔΔG” and “survival time” axes [see Additional file 7]. Each of the mutants, G41D, G41S, H46R, D101N, and D124V, was found to be considerably distinguished from each other and the rest of mutants by the hydrogen bonds inside their structure as well as by the distribution of the SEA of these hydrogen bonds [see Additional file 1]. In particular, D101N was shown to have its thermostability slightly changed as compared to the WT [16]. However, patients with this mutation live on average as little as 2 years after disease onset [16]. In contrast, the G41S mutant was significantly less stable compared to the WT, but at the same time, its clinical effects were comparable to those of D101N (patients live less than a year) [16, 62]. Of note is that patients with the G41D mutation in the same 41st position live on average 14 years [53] while its thermostability is considerably decreased [16]. Another H46R mutant differed from the others based on the fact that its thermostability was similar to the WT, though patient survival time with this mutation is 17 years [16, 53]. Metal-binding site mutant D124V had just a quarter of enzymatic activity of the WT [63]. This mutant is known to have nearly the same thermostability as the WT, but a patient with this mutation lives approximately 2 years [53]. The zinc ion in the D124V mutant was demonstrated to displace the copper ion at its binding site [64]. Of interest is that individual clusters were formed by H46R, D101N, and D124V as well when mutants were clustered by hydrogen bond STA (see Fig. 1b).

It is apparent that proportion of the results obtained employing MD or EN modelling revealed some subunit asymmetry. When detecting destructive hydrogen bonds, it was found that the count of residues forming these bonds in chain A was different from that of chain F (see Fig. 4). Similarly, when inspecting the SOD1 amino acid positions of the most averaged mechanical stiffness, one could discern the most stiff positions being slightly different in terms of their numbers across both SOD1 chains. This effect of subunit asymmetry was evident because of the non-equivalent solution of the subunits in the reference X-ray structure of SOD1 [see Additional file 8] (RMSD between both subunits over all atomic pairs was 0.467 Å) or because certain mutants exhibited varying dynamic behaviour with respect to their subunits [22, 65, 66].

When assessing the statistical significance of the difference between mutant positions and residues from metal-binding sites, dimer interface and disulfide bonds, it appeared that positions 37 and 76 were not significant [see Additional file 6]. Residues G37 and D76 are located in floppy loops that can be the cause of their decreased stiffness [13, 67]. It is worth noting that positions 37 and 76 were also outliers when analysing the correlation between survival time of patients with mutations in SOD1 and mechanical stiffness (see Fig. 6).

The importance of copper binding in supporting SOD1 structure was emphasized in this investigation when studying mechanical stiffness (see Figs. 5 and 6) and has already been mentioned in literature [68]. The value of copper ions in supporting kinetic stability was shown to be higher than that of zinc ions [68]. A number of pathogenic SOD1 mutants are known to lack copper, confirming its importance in the protein’s structure, as well [11]. In addition, the ability of mutant SOD1s to hold copper was shown to be linked with their aggregation and pathogenicity [10]. In a recent paper, SOD1 was assigned an important role in maintaining copper homeostasis within a cell, indicating that less copper binding by the protein’s mutants leads to copper accumulation, which is toxic [2]. With this, a hypothesis was put forward considering it is destabilization of metal-binding sites and the dimer interface that brings about ALS [8, 10]. Amino acid residue in the 46th position was demonstrated to take part in formation of copper-binding site, as well.

The mechanical stiffness analysis conducted in the present work based on EN modelling established that this 46th position is the most significant for distinguishing SOD1 mutants based on their pathogenicity (see Fig. 6). This might be because the mutations studied are located in the regions of the SOD1 structure that are critical, to a certain extent, to copper-binding site dynamics.

Analysis of stiffness indicated that movement of both copper ions is mutually correlated, and this can be mediated by amino acid residues, moving in a coordinated fashion and forming a compact fragment comprising the interface of the interactions between the copper ions (see Fig. 5). This fragment is also comprised of amino acid residues from the dimer interface that is known to feature prominently in ALS pathogenesis [12, 13, 15, 52]. In addition, the active centre interactions suggested could affect SOD1 regions necessary for enzymatic functioning [2, 10, 11, 68] and the protein’s stability [9–11, 68] as well as SOD1 dimerization, which is thought to be linked with both aggregation [12, 13, 15, 68] and loss of function [69].

It was found that there is a significantly positive correlation between evolutionary conservation of a given residue and its mean mechanical stiffness (see Fig. 7). Thus, positions with increased mechanical stiffness have increased evolutionary conservation, as well, suggesting their functional and/or structural importance for SOD1. The correlation between evolutionary conservation and mechanical stiffness has already been observed, for instance, when studying nesprin-1α [70].

When performing pairwise intersections (see e.g., Fig. 8) of lists with identified important positions, it was found that lists of residues forming destructive hydrogen bonds have intersections with lists of top mean mechanical stiffness residues, specifically through positions 114, 117, 149 (chain A), 114, and 149 (chain F). As it appears from the SOD1 structure, residue G114 is located in the dimer interface, and residues L117 and I149 are in close proximity to the interface according to the SOD1 sequence. Consequently, it can be stipulated that mechanical stiffness is integral to conformational changes associated with formation of destructive hydrogen bonds. Another intersection was seen between residues forming destructive hydrogen bonds and residues from the contact between SOD1 aggregates. This means that residues forming destructive hydrogen bonds are predominantly located in similar regions of the protein structure as residues forming the contact between SOD1 aggregates. This investigation showed destructive hydrogen bonds can result from or in deviations of mutant conformations from that of the WT. Moreover, residues from the contact between SOD1 aggregates involved in the formation of destructive hydrogen bonds may not only influence the direct interaction between aggregates, but also feature prominently in the formation of the pathogenic conformational states of SOD1 mutants.

The fact that the average values of the evolutionary conservation of the identified important residues is significantly different from the average evolutionary conservation of all residues in the protein means that a mechanism underlying SOD1’s structure destabilization is largely associated with the impact of its mutations on evolutionarily conserved residues.

According to our results, both SEA and STA are in robust agreement with each other. However, combining the approaches of analysis, including MD and EN modelling, to estimate the stability of hydrogen bonds can provide a better understanding of the mechanisms underlying the conformational changes induced by mutations in proteins versus using either of the approaches separately. In particular, clustering of hydrogen bonds by their STA and SEA measures produced similar results. In contrast, analysis of destructive hydrogen bonds showed that EN modelling was more efficient at uncovering a discrepancy between the SOD1 mutants and the WT than typical MD because EN modelling can account for much larger conformational fluctuations of a protein.

It is worth noting that the current study sought to analyse the stability of a single hydrogen bond in the SOD1 protein. Estimation of general stability of the protein based on hydrogen bond stabilities requires construction of additional models akin to that suggested in our previous work based on MD simulations [27]. The current results are planned for use in the future to extend our previous model for prediction of the impact of SOD1 mutations on protein stability as well as on the clinical parameters of ALS.

## Conclusions

In this investigation, modelling of the structure and dynamics of WT SOD1 and its mutants was conducted using EN. In total, 77 amino acid residues were identified that can be of importance for mutant conformations, including 42 residues forming destructive hydrogen bonds in mutants, 28 residues with the highest mean mechanical stiffness to copper ion, and 37 residues with the highest mean stiffness to all residues in the protein. These SOD1 residues identified can be responsible for the pathogenic conformations in the mutants. Taking them into account can be valuable both for understanding the molecular mechanisms of mutant pathogenicity and drug design. The results garnered in this study can aid the design of drugs that not only can prevent aggregate formation, but also repair pathogenic conformations of mutant proteins before aggregation. According to the predictions made, the regions with increased mechanical stiffness can be targeted by candidate small-molecule inhibitors which might allosterically affect SOD1 metal-binding sites.

## Additional files

Additional file 1: Table S1. Table of hydrogen bonds’ SEA. (XLSX 116 kb)

Additional file 2: Figure S1. Hierarchical clustering dendrogram based on SOD1’s SEA. (EPS 48 kb)

Additional file 3: Table S2.Table H with ${\xi}_j^m$ distribution. (XLSX 6 kb)

Additional file 4: PDB file A. SOD1 X-ray structure (PDBID: 2V0A) with b-factors being set to values of mechanical stiffness, κ_Cu,i_, between the copper ion of chain A and residues of SOD1. (PDB 342 kb)

Additional file 5: PDB file F. SOD1 X-ray structure (PDBID: 2V0A) with b-factors being set to values of mechanical stiffness, κ_Cu,i_, between the copper ion of chain F and residues of SOD1. (PDB 342 kb)

Additional file 6: Table S3.Statistical significance of difference between sets ${\overline{\kappa}}_{m_kc}$ and κ_ij_ corresponding 32 SOD1 mutant positions. (XLSX 6 kb)

Additional file 7: Figure S2. A relationship between the normalized thermostability of SOD1 mutants studied within the current work and their clinical effect expressed as survival time of an ALS patient with a corresponding SOD1 mutation. All thermostability values and survival times were taken from the literature [16]. (EPS 71 kb)

Additional file 8: Figure S3. RMSD plot between both SOD1 subunits (PDBID: 2V0A) done employing UCSF Chimera \cite {Pettersen2004}. (EPS 111 kb)

## Abbreviations

ALS: Amyotrophic lateral sclerosis
ANM: Anisotropic network model
EN: Elastic networks
MD: Molecular dynamics
PDB: Protein data bank
SEA: Stability, ensemble average
SOD1: Superoxide dismutase 1
ST: Survival time
STA: Stability, time average
WT: Wild-type

## Symbols

${SEA}_i^m$: Ensemble average stability of hydrogen bond *i* formed in mutant *m*.
*D*^*m*^: Deviation of SEA of all hydrogen bonds inside SOD1 mutant *m* from the SEA of the same bonds inside the WT.
${\xi}_j^m$: Contribution of the square of the difference between the SEA of hydrogen bond, *j*, inside a mutant *m* and the WT to the *D*^*m*^ of the mutant.
${\overline{\xi}}_j$: Average ${\xi}_j^m$ of the hydrogen bonds, *j*, calculated over the nine mutants from clusters 2, 3, and 4.
${\overline{S}}_H$: Number of hydrogen bonds {j} with ${\xi}_j^m$ being greater than 10%.
κ_ij_: Mechanical stiffness between a pair of residues in positions *i* and *j*.
κ_Cu,i_: Mechanical stiffness between the copper ion and residues of SOD1.
${\overline{\kappa}}_{m_kc}$: Average stiffness between mutant position, *m*_*k*_, and positions, *c*_*i*_, important for the protein’s structure.
$\overline{K_i}$: Average stiffness of all pairs of amino acid residue positions in SOD1 with affected position *i*
