# Correction to: selected articles from Belyaev Conference 2017: structural biology

**DOI:** 10.1186/s12900-018-0082-7

**Published:** 2018-03-21

**Authors:** Yuriy L Orlov, Ancha V Baranova

**Affiliations:** 1 Institute of Cytology and Genetics SB RAS, Novosibirsk State University, Novosibirsk Oblast, Novosibirsk, 630090 Russia; 2 George Mason University, 4400 University Dr, Fairfax, VA 22030 USA

## Correction

After publication of this supplement [1], it has been brought to our attention that there is a discrepancy between the publication date on the pdf and online formats. The date on the pdf is 6th February 2018 and online is 5th February 2018. The correct publication date is the one on the pdf, 6th February 2018.
